# Time to Recover From Daily Caffeine Intake

**DOI:** 10.3389/fnut.2021.787225

**Published:** 2022-02-02

**Authors:** Yu-Shiuan Lin, Janine Weibel, Hans-Peter Landolt, Francesco Santini, Corrado Garbazza, Joshua Kistler, Sophia Rehm, Katharina Rentsch, Stefan Borgwardt, Christian Cajochen, Carolin F. Reichert

**Affiliations:** ^1^Centre for Chronobiology, University Psychiatric Clinics Basel, Basel, Switzerland; ^2^Transfaculty Research Platform Molecular and Cognitive Neurosciences, University of Basel, Basel, Switzerland; ^3^Neuropsychiatry and Brain Imaging, University Psychiatric Clinics Basel, Basel, Switzerland; ^4^Institute of Pharmacology and Toxicology, University of Zurich, Zurich, Switzerland; ^5^Sleep and Health Zurich, University Center of Competence, University of Zurich, Zurich, Switzerland; ^6^Division of Radiological Physics, Department of Radiology, University Hospital Basel, Basel, Switzerland; ^7^Department of Biomedical Engineering, University of Basel, Basel, Switzerland; ^8^Laboratory Medicine, University Hospital Basel, Basel, Switzerland

**Keywords:** paraxanthine, withdrawal, metabolism, brain, caffeine

## Abstract

Caffeine elicits widespread effects in the central nervous system and is the most frequently consumed psychostimulant worldwide. First evidence indicates that, during daily intake, the elimination of caffeine may slow down, and the primary metabolite, paraxanthine, may accumulate. The neural impact of such adaptions is virtually unexplored. In this report, we leveraged the data of a laboratory study with *N* = 20 participants and three within-subject conditions: caffeine (150 mg caffeine × 3/day × 10 days), placebo (150 mg mannitol × 3/day × 10 days), and acute caffeine deprivation (caffeine × 9 days, afterward placebo × 1 day). On day 10, we determined the course of salivary caffeine and paraxanthine using liquid chromatography-mass spectrometry coupled with tandem mass spectrometry. We assessed gray matter (GM) intensity and cerebral blood flow (CBF) after acute caffeine deprivation as compared to changes in the caffeine condition from our previous report. The results indicated that levels of paraxanthine and caffeine remained high and were carried overnight during daily intake, and that the levels of paraxanthine remained elevated after 24 h of caffeine deprivation compared to placebo. After 36 h of caffeine deprivation, the previously reported caffeine-induced GM reduction was partially mitigated, while CBF was elevated compared to placebo. Our findings unveil that conventional daily caffeine intake does not provide sufficient time to clear up psychoactive compounds and restore cerebral responses, even after 36 h of abstinence. They also suggest investigating the consequences of a paraxanthine accumulation during daily caffeine intake.

## Introduction

Caffeine is the most frequently consumed psychostimulant worldwide. After an acute p.o. administration, 99% of the caffeine is rapidly absorbed within ~45 min (1), which brings the peak plasma concentration of caffeine at 1–2 h after the intake. The average half-life of caffeine after an acute consumption is 2.5–5 h (2), while it can be modulated by the ingested dosages, smoking, genetic variance, health status, oral contraceptives and pregnancy, and various other factors (3–6). Approximately 84% of caffeine is transformed into paraxanthine through the process of 3-methyl demethylation by the hepatic cytochrome P450 1A2 enzyme (CYP1A2) (2, 5). Following the peak concentration after a single administration of 5–8 mg/kg caffeine, plasma concentrations of caffeine decline rapidly and are surpassed by the paraxanthine levels at around 8–10 h after the intake (7). Paraxanthine has as high potency at antagonizing adenosine receptors as caffeine (8) and exerts several similar effects as caffeine, such as wake-promotion (9), psychostimulation (10), elevating blood pressure, and release of epinephrine (11). Yet, kinetics and concentrations of paraxanthine have rarely been considered when studying the physiological and cognitive outcomes of caffeine intake in humans.

Despite the common pattern of regular intake, studies on the kinetics of caffeine and paraxanthine in humans after daily caffeine intake are surprisingly scarce. In rodents, applying caffeine for 10 days in a 3 h interval leads to a smaller elimination rate constant (K_el_) of plasma caffeine concentration after 10 days of caffeine intake compared to acute administration (12). Furthermore, compared to a linear dose-response in a food-limited state, daily caffeine intake in an *ad-libitum* dietary state leads to dose-disproportional responses (i.e., the higher the dose the larger increase) in peak levels of caffeine and paraxanthine and the 24-h area under the curve (AUC_0−24_) (12). In humans, only one study compared the metabolism of caffeine in nine adults among baseline (0 mg/kg), low-dose (0.7 mg/kg), and high-dose (2 mg/kg) treatments applied every 2 h, 6 times a day for 5 days (4). The authors reported a dose-dependent deceleration of metabolism of intravenous isotope-labeled (2-'3C, 1,3-'5N2) caffeine after 3 days of treatment (i.e., the higher the dose, the slower the metabolism), and a dose-disproportional elevation in the AUC_0−24_ of paraxanthine. This study, however, included a rather small sample size with an unusually high dosage, given that the volunteer with the highest weight of 99 kg could receive up to 1,188 mg/day in the high-dose condition. It remains unclear whether the metabolism during a conventional pattern of daily caffeine intake over a longer time will adapt similarly as in this study. We consider a treatment with a unified dose in the morning, noon, and afternoon hours as more generalizable (13–15).

Although the findings from both rodents and humans summarized in the last paragraph (4, 12) consistently suggest an adapted metabolism of caffeine and paraxanthine over the course of daily caffeine intake, no physiological outcomes were available in these studies. An increase of psychoactive compounds may also multiply the impacts in brains, as *ex vivo* evidence indicates that elevating caffeine concentrations can lead to an increase in the brain-to-plasma ratio of caffeine (16). Previously, we observed a decreased gray matter (GM) in the medial temporal lobe and reduced cerebral blood flow (CBF) during daily caffeine intake, in which the larger reductions of both properties were associated with a higher accumulation of caffeine + paraxanthine. We postulated that these cerebral responses may be due to an incomplete elimination and an accumulation of the psychoactive compounds. It is still unclear, however, how fast these brain responses can be restored during abstinence when caffeine and paraxanthine can be completely eliminated.

In addition to the daily pattern of intake, genetic variations also determine the individual metabolic process and in turn modulate the development of habitual patterns of caffeine intake (5, 17, 18). In particular, the variants in CYP1A2, AHR, and CYP2A6, which are associated with lower habitual intake (19, 20), are also associated with slower metabolism of caffeine (CYP1A2 and AHR) and paraxanthine [CYP2A6; (19)]. Thus, the adaptions in the metabolism of caffeine and paraxanthine during daily caffeine intake and its physiological effects can be also modulated by the genetic traits reflected by the levels of habitual intake.

For the present report, we assumed that daily moderate-dose caffeine intake can lead to an accumulation of caffeine and paraxanthine concentrations. We leveraged salivary samples of a previous study with three within-subject conditions (21): caffeine (150 mg caffeine × 3/day × 10 days), placebo (150 mg mannitol × 3/day × 10 days), and acute caffeine deprivation (caffeine × 9 days + placebo × 1 day). We report a 43-h profile of caffeine and paraxanthine levels, the kinetics of each compound, and their association with habitual caffeine intake. Moreover, we examined the brain recovery after 36 h of caffeine deprivation when the levels of caffeine were expected to be cleared. The protocol simulated a conventional pattern of a double espresso at breakfast, lunch, and afternoon-teatime (in 3.25- and 4-h intervals, respectively).

## Methods

### Study Protocol, Participants, and Environmental Control

In a double-blind, randomized, placebo-controlled study, we included 20 clinically healthy male non-smokers, who were between 18 and 35 years of age with a body mass index between 18 and 26 kg/m^2^ and a habitual caffeine intake between 300 and 600 mg/day. The amount of habitual caffeine intake was assessed by a self-report questionnaire adapted from Bühler, Lachenmeier (22), and Snel and Lorist (23).

Each volunteer completed three conditions [orders of condition see (24)]: A placebo condition (150 mg mannitol × 3/ day × 10 days), a daily caffeine condition (150 mg caffeine × 3/ day × 10 days), and an acute caffeine deprivation (caffeine from the first administration up to day 9, then switch to placebo until the end of day 10). Note that a 9-day placebo intake can be considered to be sufficient for a washout of the remaining effects of the participants' prior caffeine intake, at least regarding possible withdrawal symptoms (25). Between each of the conditions, there was at least 1 week of recovery in which caffeine intake was not standardized, as this regular intake pattern was considered to be the status quo. Among the 20 participants (habitual caffeine level mean ± SD: 474.1 ± 107.5 mg), 10 participants habitually consumed more and 10 less than the daily dose at the start of the ambulatory part. An additional period of prior abstinence may have introduced more variance due to individual differences in the course and responses to caffeine withdrawal.

**Figure 1:**
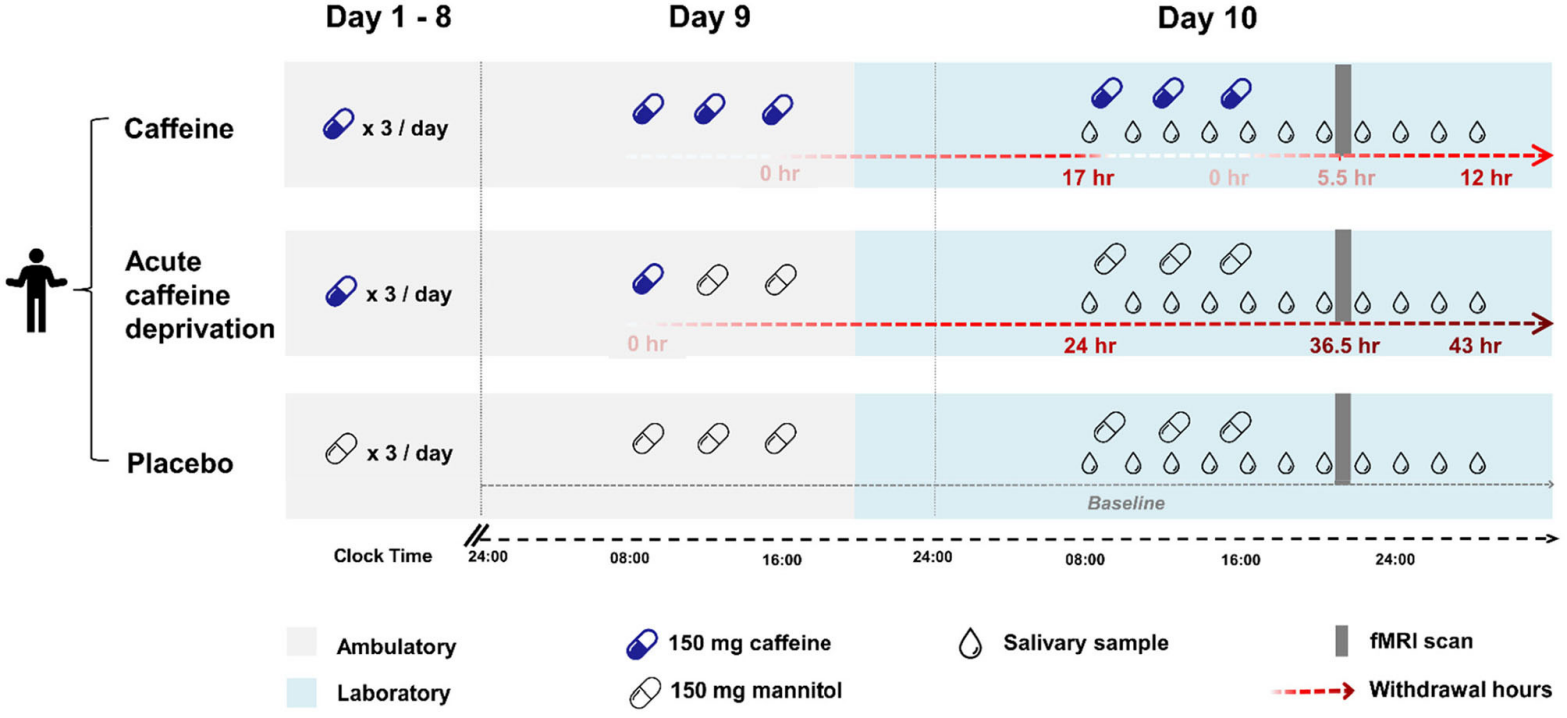
An overview of the study design. Each condition consisted of 9 ambulatory days (grey shading) and a subsequent 21-h laboratory measurement starting in the evening of day 9 (blue shading). The timing of caffeine or placebo intake is indicated by capsules (blue: caffeine, transparent: placebo), the timing of saliva collection to measure caffeine and paraxanthine levels is indicated by drops. Top: Caffeine condition. Caffeine capsules (150 mg caffeine) × 3 times/ day (at 45 min, 4 h, and 8 h after waking up) were administered throughout the 10 days. Saliva was collected repeatedly during the laboratory stay from right before the first capsule until 12 h after the latest caffeine intake. The MRI scan (grey bar) took place at 5.5 h after the latest caffeine intake. Middle: Acute caffeine deprivation. Caffeine capsules (150 mg caffeine) × 3 times/ day was administered for 8 days, followed by a switch to placebo capsules on day 9. Saliva was collected between 24 and 43 h after the latest caffeine intake. The MRI scan was scheduled at 36.5 h after the latest caffeine intake, a time window when strong withdrawal symptoms are often expressed (25). Bottom: Placebo condition. Placebo capsules (150 mg mannitol) × 3 times/ day were administered throughout the ambulatory and laboratory days. Saliva was collected at baseline through the entire laboratory phase at the corresponding time points as in the other two conditions. The MRI scan was scheduled at the same time of day as in the other two conditions.

The outcomes were assessed on day 10 for each condition. Figure 1 presents the study protocol, including the timing of the treatments, the outcome measurements, and the corresponding hours of caffeine deprivation in each condition. We scheduled the MRI scans in all three conditions for all participants at 12 h after waking up, to control for the confounding effects of different durations of wakefulness (26) and different levels of sleepiness (27) on cerebral variables.

Through the entire 10 days, participants complied with a regular sleep-wake cycle (8 h ± 30 min night-time sleep, no naps allowed) according to their habitual bedtime, which was monitored and recorded by actimetry (Actiwatch, Cambridge Neurotechnology, Cambridge, United Kingdom) and sleep diaries. Participants abstained from any other caffeine-containing diets, including coffee, tea, energy drink, soda, and chocolate, and the compliance was monitored by measuring caffeine levels in the evenings. The data of one participant's caffeine condition was excluded due to incompliance with the treatment. The laboratory environment from day 9 evening to day 10 was strictly controlled: dim light, half-supine posture (~45°), and regular dietary and lavatory times. Participants were allowed to sleep for 8 h in the night between day 9 and day 10. A digital device without time clues and access to the Internet was permitted. Social interaction was restricted to the group of experimenters.

### Data Acquisition and Analyses

#### Caffeine and Paraxanthine

In each condition, we analyzed caffeine and paraxanthine levels in 11 saliva samples, collected in 105–120 min intervals. For the quantification of caffeine, paraxanthine, theobromine, and theophylline, the collected saliva samples were analyzed using a high-performance liquid chromatography system (UltiMate 3000), coupled to a TSQ ENDURA triple quadrupole mass spectrometer (both from Thermo Scientific, Reinach, Switzerland). Ionization was performed using atmospheric pressure chemical ionization. For chromatographic separation, a ClinMass analytical ion exchange phase column (part-no MS5130, Recipe Munich, Germany) was used. Chromatography was performed with a binary gradient using 0.2% formic acid in water (mobile phase A) and methanol (mobile phase B). The gradient applied used 90% mobile phase A with a flow rate of 0.25 ml/min during 0 and 2 min, 75% mobile phase A with a flow rate of 0.3 ml/min during 2 and 4.17 min, 100% mobile phase B with a flow rate of 0.3 ml/min during 4.17 and 5.9 min, and 90% mobile phase A with a flow rate of 0.5 ml/min during 5.9 and 8.9 min. Theophylline-d6 (ClinMass AED, Recipe, Munich, Germany) was used as Internal Standard (IS) for all 4 analytes. For the quantification of the four analytes, the following mass transitions have been applied: caffeine 195.07 → 151.04 m/z, paraxanthine 181.08 → 69.26 m/z, theobromine 181.12 → 135.11 m/z, theophylline 181.08 → 124.11 m/z, and theophylline-d6 187.15 → 146.16, respectively. Twenty microliters from the clear top layer of the centrifuged saliva samples were mixed with 10 μl of IS and 100 μl of mobile phase A. After centrifugation for 5 min at 16,300 g, the clear top layer was transferred to an autosampler vial. Calibrators have been prepared in blank saliva from a caffeine-abstinent volunteer within the range of 40–8,000 ng/ml for caffeine and 20–10,000 ng/ml for the other analytes. The two quality control samples, also prepared in blank saliva from a caffeine-abstinent volunteer, had a concentration of 300 and 3,000 ng/ml for caffeine, and 150 and 1,500 ng/ml for the other analytes.

We used the samples in the caffeine condition to calculate the kinetics of caffeine and paraxanthine by peak level (Cmax), peak time (Tmax), elimination rates (K_el_), and half-lives. In addition, we characterized the accumulation of caffeine and paraxanthine by the AUC in the caffeine condition (AUCc) and the caffeine deprivation (AUCw). Furthermore, we examined the overnight residuals of each compound in the caffeine condition in the morning, right before the first treatment on day 10. This level indicated the progress of the elimination of caffeine and paraxanthine during the repetition of daily intake. Cmax was defined by the maximal level after the last caffeine administration, and the latency to Cmax (hours after the last intake) was used as *Tmax*. Due to the sampling frequency, the earliest sample collected after the last administration was roughly by 105 min, which limited the quantification of the variances of Cmax and Tmax below this threshold. A half-life was defined by the time from the peak to the time when the concentration approximates 50% of the maximal level (C_50_). AUC of caffeine and paraxanthine was calculated with the trapezoidal rule over the 11 samples separately per caffeine condition and acute caffeine deprivation.

#### Gray Matter and Cerebral Blood Flow

T1-weighted structural data were obtained with a Magnetization-Prepared Rapid Gradient Echo (MP-RAGE) sequence (1 × 1 × 1 mm^3^, TR = 2,000 ms, TE = 3.37 ms, FA = 8°) on a 3T Siemens scanner (MAGNETOM Prisma; Siemens Healthineers, Erlangen, Germany). CBF was measured by 2D Echo-Planar Imaging pulsed arterial spin labeling sequence (4 × 4 × 4 mm^3^, TR = 3,000 ms, TE = 12 ms, FA = 90). For the detailed pipeline of preprocessing and the whole-brain analyses (WBAs), please find the methods in Lin et al. (21). The current analysis adopted a region-of-interest (ROI) approach based on the previously reported caffeine-induced results in a WBA (21). The pre-defined regions are the right hippocampus for GM, and precuneus, thalamus, and basal ganglia for CBF. We extracted the mean intensity of GM (indicating both volume and density of GM) and the mean quantity of CBF in the respective ROIs in all three conditions and examined the recovery status after 36 h of caffeine deprivation compared to placebo and caffeine conditions.

#### Statistical Analysis

All the statistics were conducted on R (R core team, Vienna, Austria). To analyze the interaction effect between condition x time in caffeine and paraxanthine as well as the condition effects in the regional intensity of GM and CBF, we first estimated the coefficients of condition effects with a generalized linear mixed model by the *afex* package (28), followed by ANOVA to obtain the statistical parameters of variance analysis (F, T, and *p*-values). *Post-hoc* pairwise analysis was performed by using Tukey's multiple comparison test. The inter-individual analyses on associations between doses (including habitual caffeine intake and treatment dose) and half-lives of caffeine and paraxanthine were performed by generalized linear regressions.

## Results

### Forty-Three Hours Profiles and Pharmacokinetics

**Figure 2:**
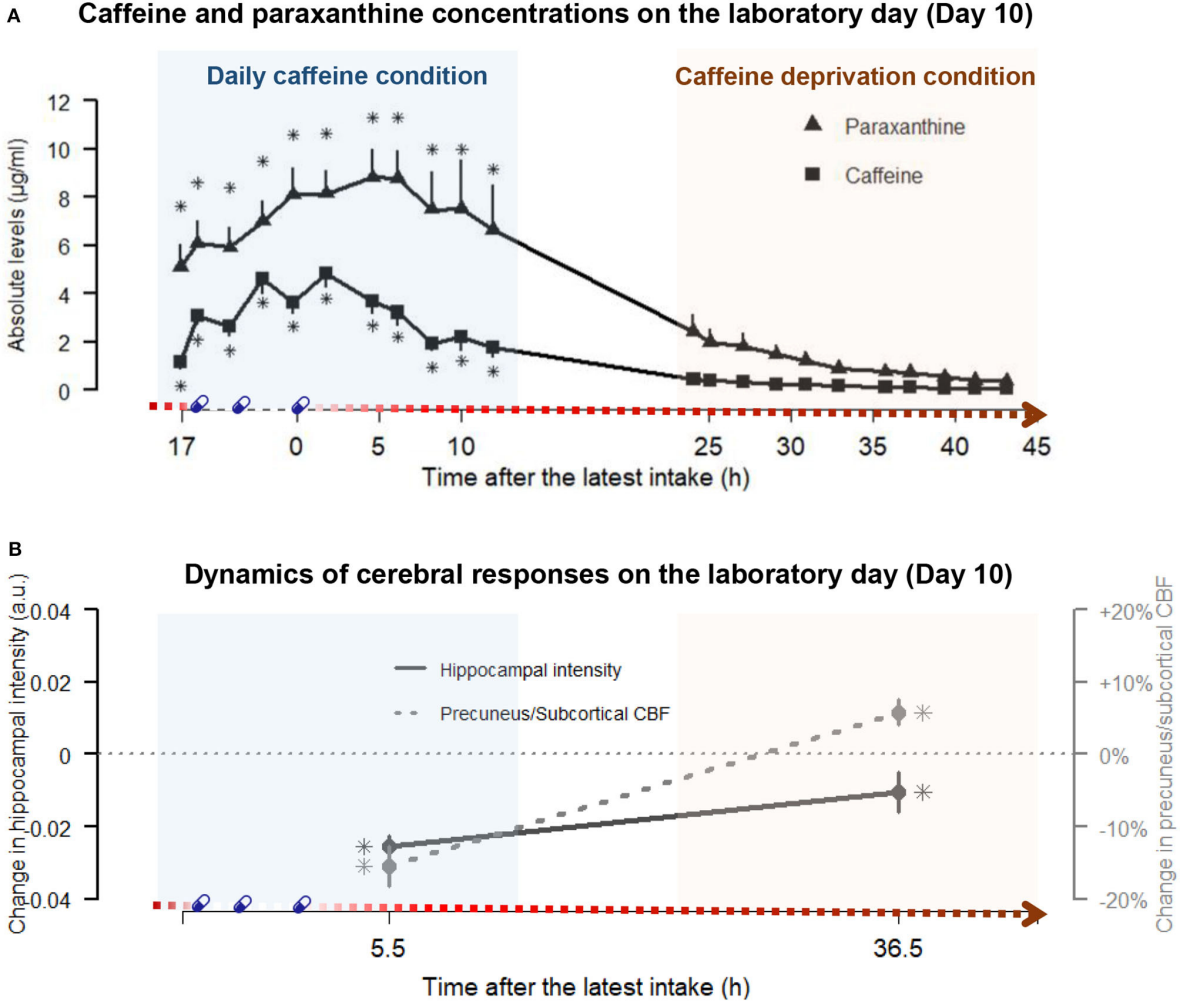
The paraxanthine and caffeine concentrations at the respective time points and the dynamics of cerebral responses on the laboratory day (day 10) in daily caffeine and caffeine deprivation conditions, respectively. **(A)** juxtaposes the profiles of caffeine (squares) and paraxanthine (triangle) in caffeine condition (blue shading, left panel) and after the caffeine deprivation (red shading, right panel) against the duration after the latest dose of caffeine on the *x*-axis. Echoing Figure 1, the gradient red arrow parallel to the x-axis schematically indicates the duration of the caffeine deprivation. The times of treatments are denoted by blue capsules. Detailed statistics are addressed in the Result section. In brief, the main effects of conditions indicated that paraxanthine levels remained elevated throughout the caffeine and the caffeine deprivation conditions compared to placebo, while caffeine levels were elevated in caffeine conditions but did not significantly differ from placebo in the deprivation. Asterisks (*) indicated the time points exhibiting significantly elevated levels compared to placebo by a *post-hoc* analysis on the significant Condition x Sample interaction. **(B)** illustrates the magnitudes of the changes in hippocampal GM intensity (straight line) and the precuneus + subcortical CBF quantity (dashed line) during daily caffeine intake and after caffeine deprivation, relative to placebo (dotted horizontal line). The asterisks indicated significant differences compared to placebo. Detailed statistics are addressed in the Results section.

Twenty volunteers (age: 26.4 ± 4.0 years; BMI: 22.7 ± 1.4 kg/m^2^; weights: 76.2 ± 8.7 kg; relative dosage of laboratory treatment: 6.0 ± 0.6 mg/kg/day; and self-reported habitual caffeine intake: 6.8 ± 2.3 mg/kg/day) completed the study. We present the 43-h profile of caffeine and paraxanthine in Figure 2, and the exhaustive data of all samples in three conditions are included in the supplement (Supplementary Table 1). The significant main effect of condition [*F*_(2, 602)_ = 618.0, *p* < 0.001] on salivary levels of caffeine indicated overall higher levels in the caffeine condition (*t* = 30.5, *p* < 0.001) compared to placebo. The overall levels of caffeine in the condition of caffeine deprivation were significantly lower compared to caffeine condition (*t* = −28.7, *p* < 0.001) but did not significantly differ from placebo (*t* = 1.7, *p* = 0.190). Indicated by the condition x sample interaction [*F*_(10, 602)_ = 9.3, *p* < 0.001], the overnight residual level of caffeine already *before* the first laboratory intake in the caffeine condition was significantly higher than in the placebo condition (see 17 h after the latest intake in Figure 2, *t* = 3.6, *p* < 0.001). Caffeine levels in the caffeine condition remained higher than in the placebo condition until 12 h after the latest intake (*t* = 5.3, *p* < 0.001).

Regarding the salivary levels of paraxanthine, we also observed a significant main effect of condition [*F*_(2, 602)_ = 400.4, *p* < 0.001]. *Post-hoc* analysis indicated that the paraxanthine levels were significantly higher in the caffeine (*t* = 25, *p* < 0.001) than in the placebo condition, while in the condition of caffeine deprivation, the levels were massively reduced (*t* = −21.7, *p* < 0.001, compared to caffeine) but still significantly higher than in placebo condition (*t* = 3.8, *p* < 0.001). Furthermore, indicated by the condition x sample interaction [*F*_(10, 602)_ = 2.0, *p* = 0.007], the overnight residual level of paraxanthine before the first laboratory intake in the caffeine condition was significantly higher than in the placebo condition (*t* = 5.4, *p* < 0.001). The paraxanthine levels remained higher than in the placebo condition after 12 h after the last intake (*t* = 6.9, *p* < 0.001).

**Figure 3:**
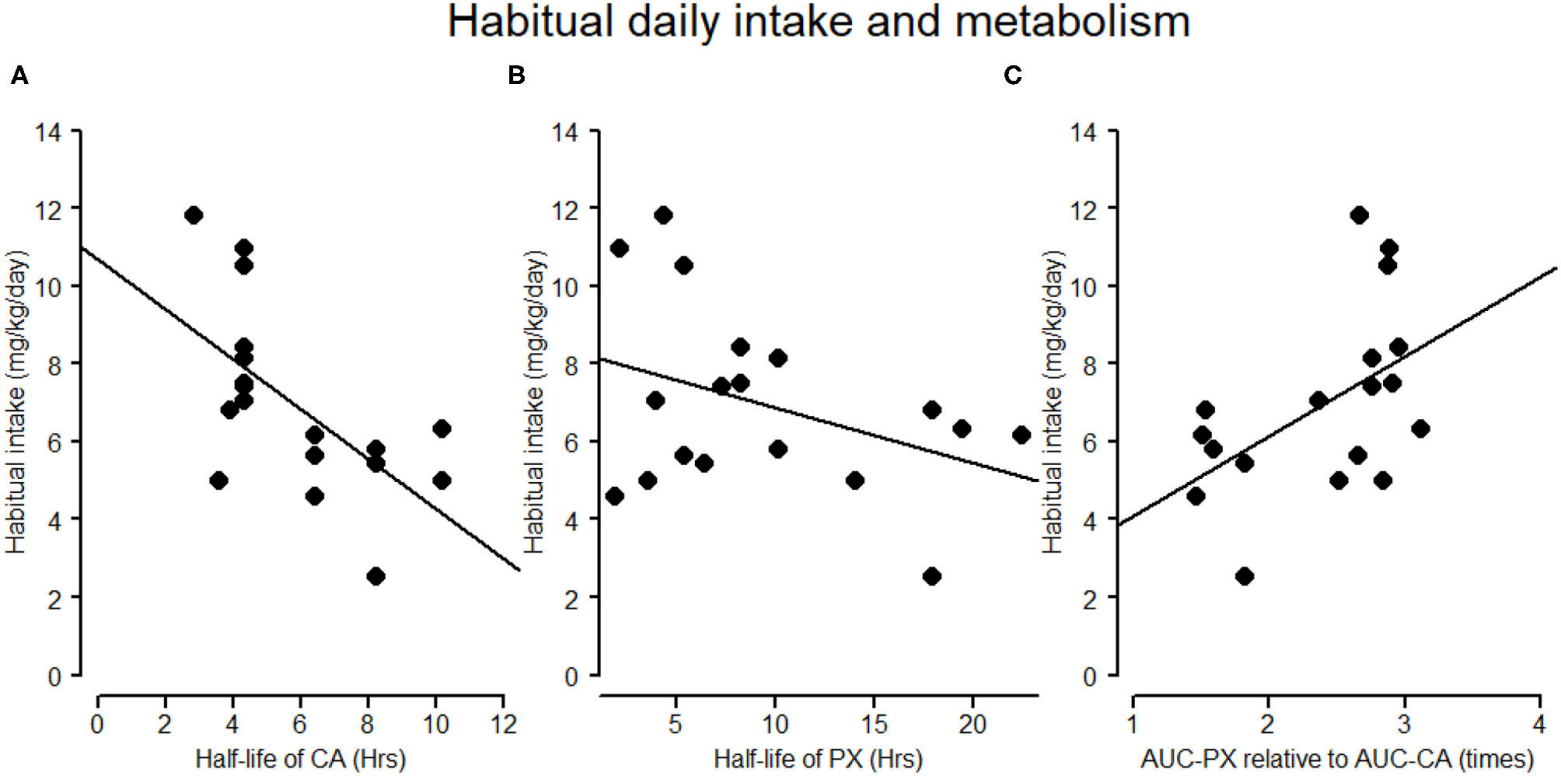
Associations of habitual daily intake and half-lives of caffeine and paraxanthine. **(A,B)** The negative association between habitual self-reported caffeine intake (mg/kg/day) and the half-life of caffeine and paraxanthine in the caffeine condition, respectively. **(C)** The association between habitual caffeine intake (mg/kg/day) and the ratio of AUC of paraxanthine (AUC-PX) to AUC of CA (AUC-CA). In **(C)** a high ratio of AUC-PX to AUC-CA indicates a larger disproportional accumulation between paraxanthine and caffeine.

As presented in Table 1, paraxanthine peaked 3.2 h later than caffeine (*t* = 3.8, *p* = 0.002) and had a 3.5 h longer half-life than caffeine (*t* = 2.7, *p* = 0.15). Furthermore, after correcting for the false discovery (reported in *q* values), the regression coefficients (Figure 3) indicated that higher habitual intake was significantly associated with shorter half-life of caffeine (β = −0.11, *p* = 0.002, *q* = 0.006) and of paraxanthine (β = −0.14, *p* = 0.04, *q* = 0.04), as well as *at trend* associated with the larger disproportionality between paraxanthine and caffeine (AUCc of PX/AUCc of CA: β = 0.05, *p* = 0.036, *q* =0.054).

**Table 1:** Peak level, peak time, half-life, and morning residuals of caffeine and paraxanthine during the caffeine condition.

	**Median (interquartile range)**		**Mean** **±SD (max.–min.)**				
	**Peak time^a^ (h)**	**Half-life (h)**	**K_**el**_**	**Peak level (μg/ml)**	**Overnight residual (μg/ml)**	AUC_**C**_ (μg*h/ml)${}^{\bar{\tau }}$	AUC_**W**_ (μg*h/ml)${}^{\bar{\tau }}$
Caffeine	1.75 (1.75^a^−1.75)	4.33 (4.33–7.79)	0.14 ± 0.05 (0.07–0.24)	5.2 ± 2.2 (3.0–11.2)	1.2 ± 1.2 (0.02–4.8)	36.1 ± 18.6 (18.2–85.0)	2.4 ± 3.5 (0.2–13.3)
Paraxanthine	4.58 (1.75^a^−6.08)	7.79 (4.60–13.10)	0.12 ± 0.10 (0.03–0.36)	11.2 ± 8.5 (5.4–42.3)	5.2 ± 4.0 (0.056–15.2)	84.8 ± 51.8 (37.4–241.8)	13.3 ± 16.8 (0.6–63.3)

a *The earliest sample after the last intake was collected at 1.75 h, which was therefore the possible minimum peak time*. *${}^{\bar{\tau }}$The AUC_C_ and AUC_W_ refer to the AUC of the 11 samples collected in the caffeine condition and acute caffeine deprivation, respectively. The coverage of the AUC is equivalent to the time from the first sample in the morning until 12 h after the last intake in the caffeine condition, and from 24 to 43 h after the last intake in the condition of acute caffeine deprivation*.

### Brain Recovery at 36.5 h of Caffeine Deprivation

As illustrated by Figure 2, the reduced hippocampal GM during daily caffeine intake reported earlier elsewhere (21) remained significantly lower after 36.5 h of caffeine deprivation compared to the placebo condition (*t*_W−P_ = −2.1, *p*_W−P_ = 0.039). The magnitude, however, was reduced after 36.5 h deprivation compared to the caffeine condition (*t*_W−C_ = 3.2, *p*_W−C_ = 0.003). On the other hand, CBF in precuneus, thalamus, and basal ganglia, which was reduced during daily caffeine intake (21), was elevated at 36.5-h caffeine deprivation compared to the placebo condition (*t*_W−P_ = 2.1, *p*_W−P_ = 0.039). In the supplement (Supplementary Figure 1), we also reported the results from the voxel-based WBA on GM and CBF after 36 h of caffeine deprivation compared to baseline.

## Discussion

The present study provides the first estimates for the kinetics of caffeine and paraxanthine and the responses of GM and CBF during and after a typical daily caffeine intake pattern [i.e., intake in the morning, at noon, and in the afternoon (13–15)]. We observed elevated residuals of paraxanthine and of caffeine carried overnight during daily intake, suggesting an accumulation over repeated daily caffeine consumption. The *at-trend* association between the habitual caffeine intake and the proportion of AUC-PX to AUC-CA indicates that people with higher habitual intake may tend to have a larger accumulation of paraxanthine during caffeine metabolism than people with a lower habitual intake. Additionally, our data allowed for the first time characterizing a recovery from daily intake after 24–43 h of caffeine deprivation, where the overall paraxanthine levels remained elevated. Finally, the caffeine-induced reduction in hippocampal GM intensity was only partially recovered by 36 h of caffeine deprivation. In contrast, CBF was significantly elevated compared to placebo. Taken together, our data suggest that conventional daily caffeine intake does not provide sufficient time for the elimination of caffeine and paraxanthine. Furthermore, even though the salivary caffeine nearly reaches a clear state, the caffeine-associated brain responses will require a longer time to be fully restored. Finally, the accumulation of paraxanthine entails a critical role in the effects of caffeine consumption during chronic intake. Given its high potency at the cerebral adenosine receptors (8), the potential effects of prolonged exposure to this xanthine and its clinical application should be further inspected in the future.

### Caffeine Metabolism, Accumulation of Paraxanthine, and Habitual Caffeine Intake: A Genetic Trait?

Daily repeated intake of caffeine is a very common phenomenon and occurs in adults more frequently as compared to acute irregular consumption of the psychostimulant after a certain phase of abstinence [patterns of regular caffeine intake, see (14)]. Besides the present data, however, the evidence on the course of human caffeine metabolism under conditions of daily intake is scarce.

With a unified dose of 150 mg × 3/day, our study had an average relative dose of 2 mg/kg × 3 times/day in a 4-h interval. The median peak time and half-life of caffeine (<105 min) in this study are very similar to the acute intake reported in earlier studies (7, 29–32). However, compared to the only available study of daily intake (4), our participants exhibited much faster metabolism in caffeine and paraxanthine, which might be due to a shorter dose interval (2 h) in their study compared to ours (4 h). The interval of 4 h was nearly equal to the half-life time of caffeine and thus may have reduced the iteration of the caffeine concentration over repeated intake.

The faster metabolism compared to the earlier study (4) might also reflect genetic traits in our participants, who had an average level of habitual intake level of 474.1 ± 107.5 mg. Several genome-wide association studies (GWAS) have discovered the link between habitual caffeine intake and caffeine metabolism through the polymorphism of CYP1A2, AHR, and CYP2A6 (17–20). Specifically, the genetic variants of CYP1A2 and AHR associated with a lower habitual caffeine intake are also associated with slow caffeine metabolism [i.e., a higher plasma level of caffeine and lower ratios of paraxanthine to caffeine (19)]. This genetic tendency is supported by our salivary data, which showed that a lower habitual caffeine intake was associated with a longer half-life of caffeine, a longer half-life of paraxanthine, as well as a lower ratio of paraxanthine to caffeine. The elevated paraxanthine levels over the entire course might also reflect the tendency to have a fast transformation of caffeine to paraxanthine in high caffeine consumers, yet without a commensurate speed for the metabolism of paraxanthine.

As our study does not include a washout period before each ambulatory part, we cannot exclude that the accumulation of paraxanthine might have been carried over from participants' prior habitual intake. This does, however, not contradict the suggested risk for an accumulated paraxanthine concentration as a consequence of habitual or daily caffeine. A dose-disproportional elevation in paraxanthine is not limited to daily intake but can also be observed when increasing the dose of intake (33).

In summary, we observed an accumulation of paraxanthine present during a pattern of caffeine intake, which imitated a realistic dose and timing of consumption. This accumulation was further intensified in people with a higher dose of habitual caffeine consumption. Paraxanthine is considered to possess lower toxicity and anxiogenesis than caffeine and other methylxanthines (9, 34, 35), while it can still exert similar effects as caffeine in wake-promotion (9), psychostimulation (10), elevated blood pressure, and release of epinephrine (11). Since paraxanthine levels are prone to be mounted over daily intake, understanding the cognitive and physiological outcomes during prolonged exposure to paraxanthine will clarify the long-term benefits and harms of daily caffeine intake.

### A Role of Habitual Caffeine Intake in the Cerebral Responses to Caffeine?

Earlier GWAS did not support the association between variants of CYP1A2 or AHR and long-term neurocognitive performance (17, 36). Nonetheless, a genetic trait for the susceptibility of the cerebral responses to caffeine intake might instead lie at the ADORA2A gene. Behaviorally, the polymorphism of ADORA2A has been linked to some caffeine-induced neuropsychological responses (37–40) and is also associated with the levels of habitual caffeine intake (37, 41). At the synaptic level, ADORA2A is responsible for not only the expression of adenosine A2A receptors (A2AR) but also the modulation of glutamatergic signaling (42) and dopaminergic receptors (38) as well as the distributions of adenosine A1 receptors (A1R) in the emotion-regulating regions, including the hippocampus (43). Adenosine A1R and A2AR play counteractive roles in the hippocampal synaptic homeostasis (44, 45), where A2AR facilitates presynaptic glutamate release while A1R modulates the postsynaptic NMDA receptors and reduces excitatory signals (44). Hence, a potential modification of A1R and A2AR expressions by a polymorphism of ADORA2A might set a predisposition for the neural responses toward the antagonist, caffeine. For instance, compared to people with C genotypes of ADORA2A, ADORA2A T/T allele carriers had higher average levels of habitual caffeine intake and a higher susceptibility to the anxiogenic effect of caffeine as well as an increased expression of A1R in frontal, hippocampal, and entorhinal cortices (43). As moderate-to-high caffeine consumers, our participants might bear the genetic propensity to have stronger responses toward A1R antagonism by caffeine, which might pose a disposition to hippocampal plasticity or excitotoxicity. Future studies are therefore recommended to include a wider range of habitual caffeine consumers or collect genetic information for better precision.

To date, there is no direct evidence on the link between ADORA2A and caffeine-induced hippocampal plasticity or CBF in healthy humans. It has been found, however, that the variants of ADORA2A were associated with the degeneration of hippocampal gray matter in both schizophrenic patients (46) and aging populations with cognitive decline (42). Adding to this point, the frequently reported association between lifetime caffeine intake and a reduced risk for cognitive decline (47–50) or neurodegeneration (51–53) could be driven by a common genetic factor. Our results ad to this point that at least in a certain population–potentially consumers who naturally accommodate to moderate habitual caffeine consumption–a daily intake of moderate-dose caffeine might instead increase the risk for a reduction in hippocampal gray matter.

### Do We Sufficiently Recover During Daily Caffeine Intake?

Withdrawal responses commonly occur after discontinuing regular caffeine intake. While our reports elsewhere have discussed in detail the implications of cerebral effects of caffeine (21, 54), the current analyses further added that these changes might take longer than the interval of day-to-day intakes to recover. We corroborated the caffeine cessation-induced vasodilation by the elevated CBF (55–60). An increased CBF, which could be attributed to the enhanced adenosine-modulated vasodilation after chronic caffeine exposure (61–63), was also frequently observed during increased sleep pressure, such as in the evening compared to morning (26), after sleep restriction (64), and after sleep deprivation (26). In line with the literature, the cognitive responses in the same volunteers reported elsewhere, including reduced vigilance, increased sleepiness, and enhanced sleep depth (24, 65), confirmed that the participants were experiencing a solid withdrawal state.

Responses toward an acute caffeine deprivation can manifest as a restoration of adapted neural functioning when caffeine intake is ceased. The molecular mechanism of such adaptations includes a mounting concentration of extracellular endogenous adenosine (61) and upregulated adenosine receptors (66–68). Both mechanisms can lead to a surge of cognitive and physiological reactions through the strengthened adenosine binding. These reactions include fatigue, reduced concentration, mood disturbance, headache, as well as increased vasodilation as addressed (25, 69, 70). Intense withdrawal symptoms are usually perceived around 20 and 50 h after the last regular intake and, in extreme cases, can last maximal 9 days (25). The earliest observed neurovascular responses to caffeine deprivation in existing evidence were at 21 h (58).

While combating withdrawal symptoms are often the reason to consolidate the daily repeated consumption of caffeine (71), the typical daily repeated caffeine intake, however, is unlikely to provide enough time for a full “withdrawal-driven restoration.” The first evidence comes from our observation in the overnight residuals of caffeine and paraxanthine levels, which were measured at 17 h after the last intake. Furthermore, during the 24–43 h of caffeine deprivation when the caffeine levels were nearly cleared, the paraxanthine levels remained elevated. In other words, a repetition of intake shorter than this time window is most likely to be insufficient for the full elimination of both caffeine and paraxanthine. The prolonged presence of high levels of such adenosine antagonists may impede the neural homeostasis dependent on the activations of adenosine receptors. In neurogenesis of adult rodents, the presence of a similar dose of caffeine during sleep has been shown to suppress the cell proliferation in the hippocampus [(72); Dose conversion: 10 mg/kg in rodents were estimated to be equivalent to 250 mg/ 70 kg in humans (71)]. The concurrence of a reduced hippocampal GM intensity and an incomplete caffeine elimination, together with a GM recovery after being deprived of caffeine, underscore the importance of a sufficient cessation period. The overcompensation of the CBF response after the caffeine deprivation, again, points to a longer time, perhaps some consecutive days, for full recovery. Future studies should confirm this postulation with a design which allows full elimination caffeine and paraxanthine as well as an observation of the response in cerebral morphology.

## Limitations

This study bears a few limitations which should be carefully taken into account. First, as discussed earlier, the current analysis leveraged the data collected in a study without a washout period before the start of ambulatory treatment. This may limit the precise attribution of observed metabolic outcomes to the doses and durations of laboratory treatment. However, it did not completely compromise our interpretation of the impacts of the habitual intake, as the variations of the habitual intake levels were restricted by the selection criterion and are comparable to laboratory caffeine intake (300–600 mg/day, average 474.1 ± 107.5 mg/day). Second, one might consider the dose administered in our study (150 mg 3 times daily) to be rather high. Earlier studies reported an average caffeine intake per capita varying from 16 to 400 mg/day per capita worldwide (71, 73). However, this number can be underestimated in regular consumers by taking the non-consumers to average (73). Furthermore, while consumption of coffee and tea were the primary target of many large-scale country-wise surveys, some other caffeinated products are often missing, such as cola, chocolate, and energy drinks, which could also lead to an underestimation. Thus, the outcomes in our study derived from 450 mg of daily intake may be particularly of interest to the regular consumers, especially those who may consume multiple types of caffeinated products. On the other hand, one might consider that quantifying habitual caffeine levels by self-reports may not be accurate, yet earlier studies have repeatedly reported generally good reliabilities between self-report caffeine intake and salivary concentrations of caffeine and paraxanthine unless the self-report amount is >600 mg/day (74, 75). In addition, one might consider our sample size to be relatively small despite a within-subject design. Our sample size calculations were indeed based on reported effect sizes of caffeine intake mainly on sleep-wake regulatory indices. Furthermore, we attempted to reduce the variance derived from the sex differences by only including male participants. Thus, the generalizability of the outcomes to female caffeine consumers is limited. Finally, due to a convenient advantage taken from an existing study, the sampling timing of salivary concentrations may not be optimal, especially a relatively later first sample after the caffeine intake (~105 min). This can potentially bias the calculation of kinetics. It is of importance to note that the kinetics of caffeine are rather comparable to the earlier evidence as discussed previously. Paraxanthine, on the other hand, did not suffer from this issue, as its concentration peaked much later than the earliest sample after the caffeine intake. Finally, future studies may continue the dynamic perspective and precisely illustrate dose-dependent changes in the metabolism of caffeine and paraxanthine from both salivary and plasma samples in healthy populations.

## Significance

Caffeine is consumed on a daily basis among 80% of the worldwide population (73). It is of importance to beware that daily consumption, even merely in the daytime, can accumulate exposure to the psychostimulant and prevents the body from full recovery. The in-progress recovery from a reduced GM and the elevated CBF after 36 h of caffeine cessation entails a longer time required for full restoration than the conventional repetition of daily intake. On the other hand, the accumulation in paraxanthine underscores the importance to investigate its cognitive and physiological effects, which may be responsible for long-term outcomes of chronic exposure to caffeine. Methodologically, the adapted metabolism also suggests a careful consideration to translate acute effects of caffeine onto daily usage. Finally, the responses of GM and CBF in both the caffeine condition and after 36 h of caffeine deprivation emphasize the importance of restricting caffeine intake when studying cerebral morphometry and neurovascularactivities.

## Data Availability Statement

The raw data supporting the conclusions of this article will be made available by the authors, without undue reservation.

## Ethics Statement

The studies involving human participants were reviewed and approved by Ethics Committee Northwest/Central Switzerland (approval reference: EKNZ 2016-00376). The patients/participants provided their written informed consent to participate in this study.

## Author Contributions

Y-SL: data acquisition, analysis, and drafting the manuscript and figures. JW: data acquisition. H-PL: conception of the study. FS: design of the study. CG: data acquisition and medical consultant. JK and SR: measurement of salivary samples. KR: measurement of salivary samples and supervisor. SB: design of the study and supervisor. CC: conception and design of the study. CR: conception and design of the study and drafting the manuscript. All authors contributed to the article and approved the submitted version.

## Funding

This study was supported by Swiss National Science Foundation (grant 320030-163058).

## Conflict of Interest

The authors declare that the research was conducted in the absence of any commercial or financial relationships that could be construed as a potential conflict of interest.

## Publisher's Note

All claims expressed in this article are solely those of the authors and do not necessarily represent those of their affiliated organizations, or those of the publisher, the editors and the reviewers. Any product that may be evaluated in this article, or claim that may be made by its manufacturer, is not guaranteed or endorsed by the publisher.
